# Laparoscopic repair of femoral hernia involving the bladder with coexisting indirect inguinal hernia in a young man: a case report

**DOI:** 10.1186/s40792-021-01334-0

**Published:** 2021-12-07

**Authors:** Ryoma Yokoi, Shigetoshi Yamada, Yuji Hatanaka, Hiroki Kato

**Affiliations:** Department of Surgery, Tajimi City Hospital, 3-43, Maebata, Tajimi, Gifu 507-8511 Japan

**Keywords:** Femoral hernia, Hernia involving the bladder, Transabdominal preperitoneal repair

## Abstract

**Background:**

Bladder hernias are rare conditions that are difficult to diagnose preoperatively; many cases are diagnosed intraoperatively or postoperatively due to bladder injury. Most bladder hernias are direct inguinal hernias that involve the bladder in obese men older than 50 years old. We describe a rare case of a left femoral hernia involving the bladder in a young man.

**Case presentation:**

A 32-year-old man with a bulge in the left inguinal region underwent laparoscopic transabdominal preperitoneal repair. Laparoscopy revealed a left indirect inguinal hernia. When the preperitoneal space was dissected toward the Retzius space along the vesicohypogastric fascia, the bladder was found to be protruding into the femoral ring and adhere to the hernial orifice severely. The bladder was reduced carefully without causing injury. After dissection, we repaired the left myopectineal orifice with a mesh. The patient was discharged on postoperative day 1 without complications. No recurrences or symptoms were noted at the 12-month follow-up.

**Conclusions:**

A femoral hernia involving the bladder in a young man is rare. This case demonstrated that dissection along anatomical landmarks is important for preventing injuries to the bladder because even young men may have bladder hernias.

## Background

Groin hernias usually contain intra-abdominal viscera surrounded by the peritoneum, and extraperitoneal organs are not typically contained. Bladder hernias occur by sliding through the inguinal or femoral canal due to the hernia sac pulling the bladder. Most bladder hernias are direct inguinal hernias that involve the bladder. Inguinal bladder hernias usually occur on the right side in obese men older than 50 years old, and comprise 0.5–4.0% of all inguinal hernias [1–3]. Femoral hernias are common on the right side in women older than 50 years old. Femoral hernias involving the bladder make up approximately one-fourth of all bladder hernias in the groin [4, 5]. Bladder hernias are rare conditions that are difficult to diagnose preoperatively; many cases are diagnosed intraoperatively or postoperatively due to bladder injury [6]. Therefore, it is important to prevent bladder injury by keeping in mind the coexistence of bladder hernias during the preperitoneal mesh repair procedure such as laparoscopic transabdominal preperitoneal repair (TAPP). We describe a rare case of a left femoral hernia involving the bladder in a young man.

## Case presentation

**Fig. 1:**
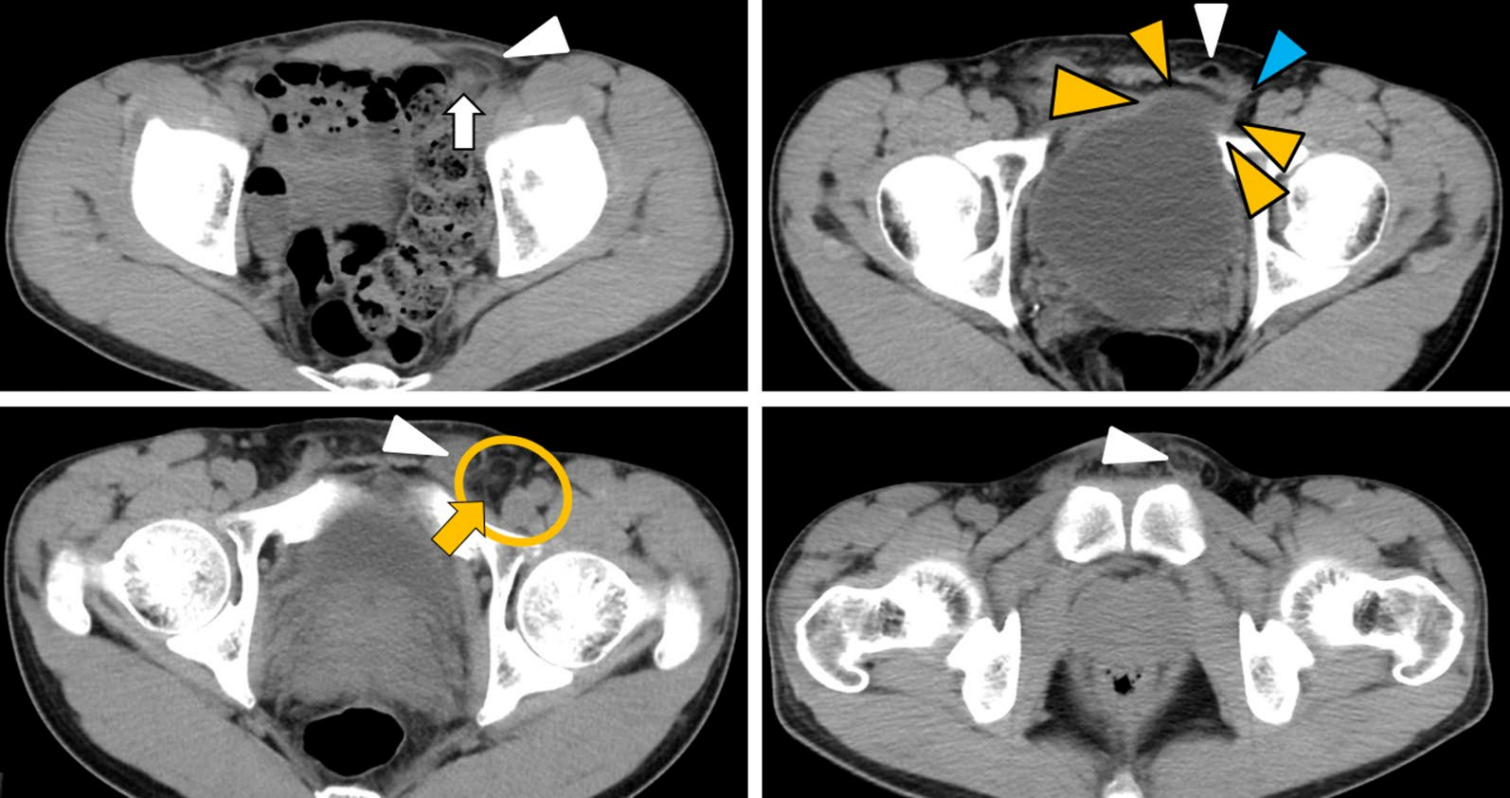
Computed tomography (CT) performed five years before admission. CT showed a left indirect inguinal hernia (white arrowheads) which protruded from the outside of the inferior epigastric vessels (white arrow). A portion of the bladder (yellow arrowheads) was pulled to the left femoral ring (blue arrowhead). The small adipose tissue (yellow arrow) was observed in the left femoral canal (yellow circle)

A 32-year-old man was admitted to our hospital because of a bulge in the left inguinal region. He had no pain and no urinary symptoms. Physical examination revealed a non-tender, reducible bulge in the left inguinal region. His body mass index (BMI) was 19.0 kg/m^2^. Laboratory data were all within the normal range. Computed tomography (CT) performed 5 years before admission for other disease showed a left indirect inguinal hernia. In addition, a small protrusion of the adipose tissue into the left femoral canal and a portion of the bladder pulled to the left femoral ring were observed retrospectively (Fig. 1). Preoperative imaging studies were not performed. Based on a diagnosis of left indirect inguinal hernia, we planned for TAPP.

**Fig. 2:**
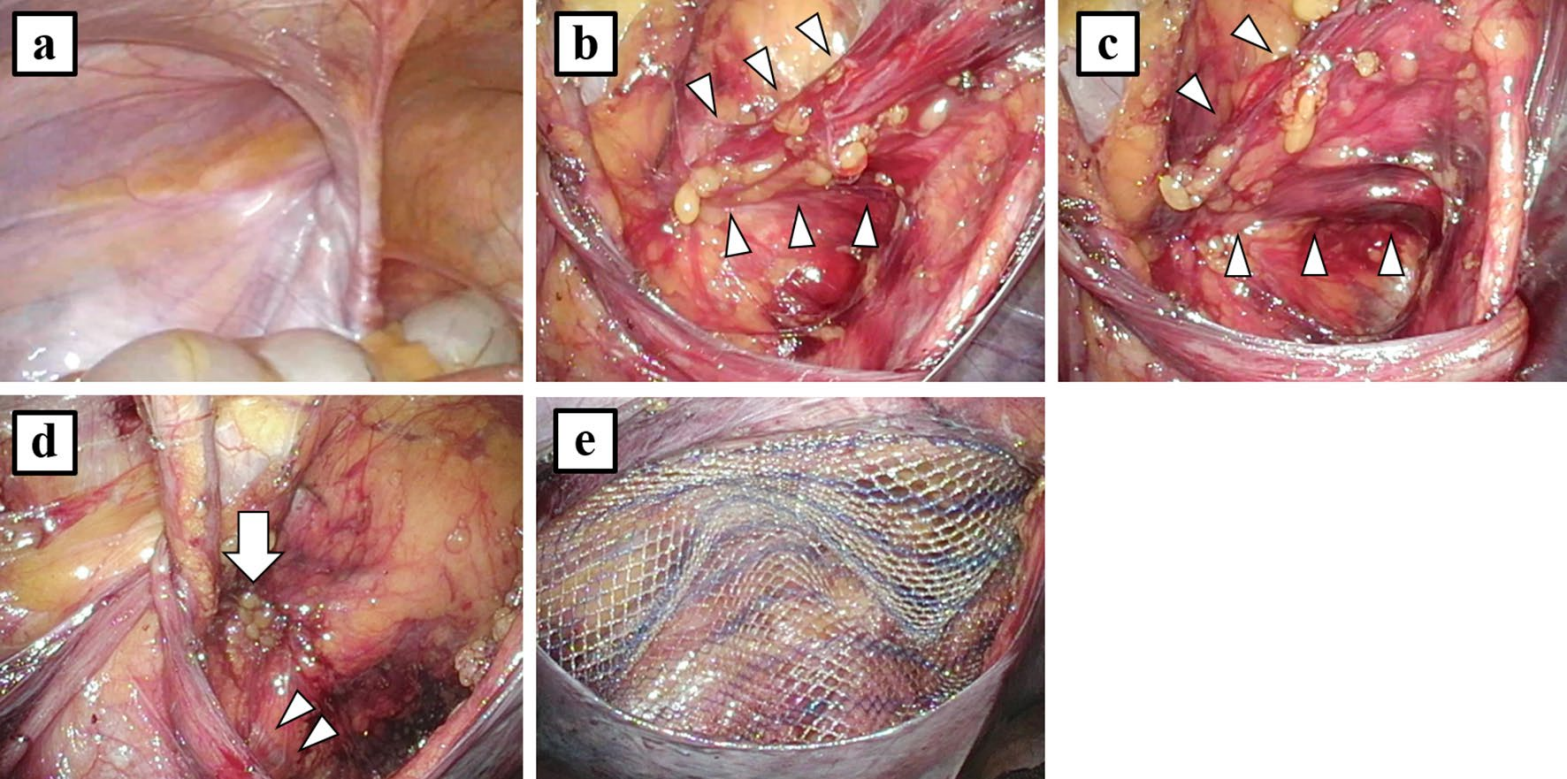
Operative findings. **a** Laparoscopy and intraperitoneal observation revealed a left indirect inguinal hernia. There were no other peritoneal indentations. **b** A mass covered with the vesicohypogastric fascia (arrowheads) was pulled into the femoral ring in the preperitoneal space. **c** The mass was confirmed to be a portion of the bladder (arrowheads); the mass expanded when saline was injected through the urinary catheter. **d** The protruding bladder was reduced carefully from the femoral ring (arrow). An aberrant obturator artery was observed (arrowheads). **e** The left myopectineal orifice was repaired with a mesh

The patient was placed in the supine position under general anesthesia. A urinary catheter was inserted before the operation. A 10-mm port was placed for the laparoscope at the umbilicus and two 5-mm ports were placed at each flank. Laparoscopy and intraperitoneal observation revealed a left indirect inguinal hernia with sliding of the surrounding tissue. There were no other peritoneal indentations (Fig. 2a). We incised the peritoneum and dissected the preperitoneal space toward the Retzius space along the vesicohypogastric fascia. We found a mass covered with the vesicohypogastric fascia that was pulled into the femoral ring (Fig. 2b). The mass was confirmed to be a portion of the bladder; the mass expanded when we injected saline through the urinary catheter (Fig. 2c). We first dissected the protruding bladder all around from the preperitoneal tissue sliding with the bladder because the protruding bladder had developed severe adhesion to the hernial orifice. Then we reduced the protruding bladder carefully without causing injury. The length of the herniated bladder in the femoral canal was about 1 cm. The diameter of the femoral ring was 1 cm. An aberrant obturator artery was observed (Fig. 2d). After additional dissection, we repaired the left myopectineal orifice with ULTRAPRO Partially Absorbable Lightweight Mesh (15 × 10 cm, Johnson & Johnson, Cincinnati, OH) (Fig. 2e). Operative time was 101 min. The patient was discharged on postoperative day 1 without complications. No recurrences or symptoms were noted at the 12-month follow-up.

## Discussion

We report a case of a femoral hernia involving the bladder with a coexisting indirect inguinal hernia in a young man treated with TAPP. Bladder hernias range from small to massive protrusions. Massive protrusions represent < 1% of bladder hernias [7]. Large hernias may present with two-stage micturition or non-specific symptoms such as urinary frequency, urgency, hematuria, or nocturia. In contrast, small hernias, which make up most bladder hernias, are usually asymptomatic [3, 8, 9]. Risk factors for bladder hernias include aging, obesity, urinary outlet obstruction, and loss of bladder tone with weakness of the supporting structures [3, 9]. Ultrasonography and radiological investigations such as CT, magnetic resonance imaging, or cystography are useful for preoperative diagnosis and avoiding intraoperative bladder injury [9]. There have been increasing reports recently that large bladder hernias could be diagnosed preoperatively and bladder injury could be avoided. However, imaging is often not performed for common groin hernias, and it is difficult to make a preoperative diagnosis of bladder hernias, especially asymptomatic small ones [10]. In the present case, CT performed 5 years before admission showed an indirect inguinal hernia, and we were not aware of a small protrusion of the adipose tissue into the left femoral canal and a portion of the bladder pulled to the left femoral ring before the operation. We had no suspicion of bladder hernia because these risk factors were not present, and preoperative imaging studies were not performed. CT is not commonly performed on young patients with groin hernias because of radiation exposure. We should have performed ultrasonography instead even if it was difficult to diagnose a small bladder hernia.

Bladder hernias are classified anatomically into three subtypes based on their relationship to the peritoneum: paraperitoneal, extraperitoneal, and intraperitoneal. The paraperitoneal type is the most common [8, 11]. Our case was the extraperitoneal type, which is the most infrequent type. The extraperitoneal type is often asymptomatic because the mean value of the prolapsed bladder length was 0.8 cm and was shorter than that of the other two types [11].

The primary etiology of femoral hernias is a congenitally narrow posterior inguinal wall attachment onto Cooper’s ligament, which results in an enlarged femoral ring. A secondary cause is increased intra-abdominal pressure, which forces preperitoneal fat into a congenitally large femoral ring [12]. In our case, CT performed 5 years before admission showed a small protrusion of the preperitoneal fat into the left femoral canal and a portion of the bladder pulled to the left femoral ring, suggesting a pre-condition of bladder hernia. His indirect inguinal hernia was accompanied by sliding of the surrounding tissue. Therefore, congenital anatomical variations and acquired factors might have resulted in a femoral hernia. In addition, the bladder was pulled laterally due to sliding of the preperitoneal tissue toward the indirect inguinal hernia orifice. The bladder had eventually protruded into the femoral ring.

We searched PubMed and Japanese literature, and summarized 10 cases of femoral hernia involving the bladder including our case (Table 1) [10, 13–20]. The 10 cases comprised five men and five women. In all cases excluding ours, the patients were older than 50 years old. There were both obese and slender patients. Fewer hernias were detected on the left side than on the right side. Four patients had urinary symptoms. The number of paraperitoneal and extraperitoneal type were almost same. There was no intraperitoneal type. Only two cases including ours were treated with TAPP, and the others were treated by open surgery. Two cases including ours were associated with indirect inguinal hernias. Groin hernias involving the bladder can be combined with other groin hernias on the same side [11]. Particularly for the extraperitoneal type, the bladder hernia and the hernial orifice cannot be identified through intraperitoneal observation. Therefore, during laparoscopic hernia repair, it is important to dissect bluntly and meticulously while checking for anatomical landmarks since there might be a bladder hernia. We should identify the vesicohypogastric fascia and dissect the protruding bladder all around along the vesicohypogastric fascia without damaging it to avoid misidentification of the bladder as a hernia sac or adipose tissue. Injecting saline or indigo carmine through a urinary catheter reveals the boundary between the bladder and the hernial orifice [11]. Bladder injury can be confirmed because of the leakage. Since incarceration occurs frequently with femoral hernias, we should consider the combination of manual reduction, placing an additional port, converting to open surgery, or resection of the protruding bladder when the reduction of the bladder hernia is difficult due to adhesion [3, 10]. In addition, an aberrant obturator artery, which originates from the external iliac artery instead of the internal iliac artery, can pass just near the femoral ring [21, 22]. We need to be aware of possible vascular variations and their close proximity to the femoral ring to avoid dangerous hemorrhage during dissection of the herniated bladder. There have been several reports of laparoscopic repair of inguinal bladder hernia recently which stated usefulness of laparoscopic surgery [9]. In our case, laparoscopic surgery was useful for bladder hernia, even femoral hernia, because it had good visibility and could reduce the risk of misidentification of the bladder, bladder injury, or dangerous hemorrhage.

**Table 1:** Reported cases of femoral hernia involving the bladder

	Author	Year	Age	Sex	BMI (kg/m^2^)	Side	Urinary symptom	Subtype of bladder hernia	Other hernia	Surgical approach
Case 1	Buchholz et al. [13]	1998	72	Male	N/A	Right	Dysuria, groin pain during voiding	N/A	None	N/A
Case 2	Sakano et al. [14]	1998	61	Female	20.1	Right	None	Extraperitoneal type	None	Lower midline incision
Case 3	Kaneko et al. [15]	2005	73	Male	28.1	Left	None	Extraperitoneal type	None	Oblique incision, extraperitonealy
Case 4	Reeve et al. [16]	2009	87	Female	N/A, slender	Right	Urinary frequency	Extraperitoneal type	None	Lower midline incision for small bowel obstruction
Case 5	Matsuta et al. [17]	2009	65	Male	25.3	Right	None	Extraperitoneal type	None	Pfannenstiel incision, extraperitonealy
Case 6	Omari et al. [18]	2013	59	Male	32	Right	Dysuria, nocturia, urinary urgency	Paraperitoneal type	Indirect inguinal hernia	Pfannenstiel incision, extraperitonealy
Case 7	Harada et al. [19]	2013	83	Female	20	Right	None	Paraperitoneal type	None	Oblique incision, extraperitonealy
Case 8	Fujinaga et al. [10]	2018	91	Female	18.6	Right	None	Paraperitoneal type	None	TAPP
Case 9	Biswas et al. [20]	2020	65	Female	N/A	Right	Hematuria	Paraperitoneal type	None	Oblique incision, extraperitonealy
Case 10	Our case	2021	32	Male	19	Left	None	Extraperitoneal type	Indirect inguinal hernia	TAPP

*BMI* body mass index, *N/A* not available, *TAPP* laparoscopic transabdominal preperitoneal repair

## Conclusions

We experienced a rare case of a left femoral hernia involving the bladder with a coexisting indirect inguinal hernia in a young man treated with laparoscopic surgery. This case demonstrated that dissection along anatomical landmarks is important for preventing injuries to the bladder because even young men may have bladder hernias.

## Data Availability

The datasets supporting the conclusions of this article are included within the article.

## Abbreviations

TAPP: Laparoscopic transabdominal preperitoneal repair
BMI: Body mass index
CT: Computed tomography
